# Pharmacological Interventions That Target Biological Aging

**DOI:** 10.1093/geroni/igaa057.2980

**Published:** 2020-12-16

**Authors:** Matt Kaeberlein

**Affiliations:** University of Washington, Seattle, Washington, United States

## Abstract

There is a high level of interest in drugs that may delay or even reverse the functional declines and disease risks that accompany biological aging. Several interventions have been shown to improve age-related outcomes and increase lifespan in laboratory animals by targeting the hallmarks of aging. A number of these small molecules are being clinically evaluated for age-related indications, including mTOR inhibitors such as rapamycin, the anti-diabetic drug metformin, and senescent-cell clearing senolytics. Others are being marketed to consumers outside of the federal regulatory process as “anti-aging” natural products with little information about safety or efficacy. Here I will provide an overview of the current state of “anti-aging drugs” with an emphasis on potential mechanisms of action and evaluation of the existing pre-clinical and clinical data.

